# Region-Oriented and Staged Treatment Strategy in Reconstruction of Severe Cervical Contracture

**DOI:** 10.1371/journal.pone.0122669

**Published:** 2015-04-09

**Authors:** Xusong Luo, Fei Liu, Xi Wang, Qun Yang, Shoubao Wang, Xianyu Zhou, Yunliang Qian, Jun Yang, Lawrence Scott Levin

**Affiliations:** 1 Department of Plastic and Reconstructive Surgery, Shanghai Ninth People’s Hospital, Shanghai Jiao Tong University Medical School, Shanghai 200011, China; 2 Department of Orthopaedic Surgery, Hospital of the University of Pennsylvania, Philadelphia, Pennsylvania, United States of America; University of Louisville, UNITED STATES

## Abstract

**Introduction:**

Severe cervical contracture after burns causes obvious impairment of neck movement and the aesthetic silhouette. Although various surgical techniques for treatment have been described, there is not a definitive strategy to guide treatment. Over the past 6 years, we have been utilizing a region-oriented and staged treatment strategy to guide reconstruction of severe cervical contracture. Satisfactory results have been achieved with this strategy.

**Methods:**

The first stage of treatment focuses on the anterior cervical region and submental region. Procedures include cicatrix resection, contracture release, division and elevation of the platysma to form two platysma flaps, and skin grafting. Three to six months later, the second stage treatment is performed, which localize to the mental region. This includes scar resection, correction of the lower lip eversion, and reconstruction with free (para)scapular skin flap. Three subtypes of cervicomental angle that we proposed were measured as quantitative tool for evaluation of the reconstruction.

**Results:**

24 patients who completed the treatment were reviewed. By the 3rd postoperative month, their CM angles changed significantly: the soft tissue CM angle was reduced from 135.0° ± 17.3° to 111.1° ± 11.3°, the osseous CM angle increased from 67.1° ± 9.0° to 90.5° ± 11.6° and the dynamic CM angle increased from 21.9° ± 8.7° to 67.4° ± 13.1°. 22 in 24 (91.7%) of these patients gained notable improvement of cervical motion and aesthetic contour.

**Conclusions:**

Our results suggest that the region-oriented and staged treatment strategy can achieve satisfactory functional and aesthetic results, combining usage of both skin graft and skin flap while minimizing the donor site morbidity.

## Introduction

Cervical contractures most often result from burns caused by flame, scalding, chemical and electrical current. Severe contracture results due to damage to deep tissues of extensive area including lower lip, chin, neck, and chest, the disfigured patient has significant functional and aesthetic deficits.

Treatment of severe contracture is always challenging for the reconstructive surgeon [1, 2]. Although various surgical techniques have been described [3, 4, 5, 6], no definitive consensus was formed to guide the treatment. Upon the choice of tissue type for covering the wound after release of cervical contracture, should skin graft or skin flap be used? One tissue type for the whole cervical region or only part of it? There has been different views and even controversy [7, 8, 9].

To our opinion, distinctive characteristics of various cervical regions necessitate different choice of tissue type. In recent years our treatment team has developed a region-oriented and staged treatment strategy for severe cervical contracture. The purpose of this study is to describe the outcomes of application of the strategy in patients with severe cervical contractures using the CM angle as an objective measure.

## Clinical Data and Methods

A retrospective study is designed to collect and analyze the data of 26 patients (19 male and 7 female ranging between 19 and 58 years of age) affected by severe cervical contractures. They were treated between 2007 and 2012. These includes 13 cases of flame burns, 9 cases of scalding and 4 cases of chemical burns. The interval between first injury and treatment in our institution ranged from 6 months to 20 years.

This study is granted with the approval from Medical Ethics Committee of Shanghai Ninth Hospital. The individual in this manuscript has given written informed consent (as outlined in PLOS consent form) to publish these case details.

### CM angle (cervicomental angle) measurement & statistical analysis

**Fig 1:**
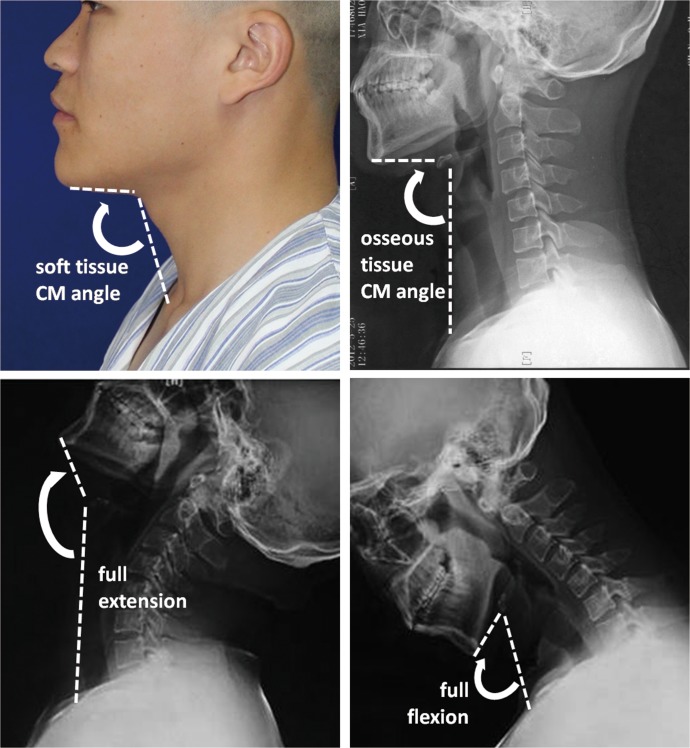
Measurement of three subtypes of CM angle. *(Above*, *left)* soft tissue CM angle (aesthetic CM angle); *(Above*, *right)* osseous CM angle (functional CM angle); *(Below)* osseous CM angles when neck in full extension and full flexion, their D-value is dynamic CM angle.

Patients were maintained in an erect position, and only movement of the cervical spine was permitted. Lateral photograph and radiograph were taken for CM angle measurement with Image-Pro Plus 6.0 (Media Cybernetics, USA) (Fig 1).

#### Soft tissue CM angle

the angle formed by three soft-tissue landmarks—Mes (menton of soft tissue), skin projective points of hyoid bone and supersternal notch. Pictures were taken with the Frankfort horizontal plane positioned parallel to the floor.

#### Osseous CM angle

the angle formed by three bony landmarks—Me (menton), hyoid bone and jugular notch of sternum when the Frankfort horizontal plane positioned parallel to the floor.

#### Dynamic CM angle

The difference (D-value) of osseous CM angles in full cervical extension and full cervical flexion respectively.

Paired-sample t-test (SPSS 12.0, USA) was used to determine whether treatment changed three subtypes of CM angle. The statistical significance was established at *p* = 0.05.

### The first stage treatment

Scar resection, and thorough contracture release of neck was performed first.

**Fig 2:**
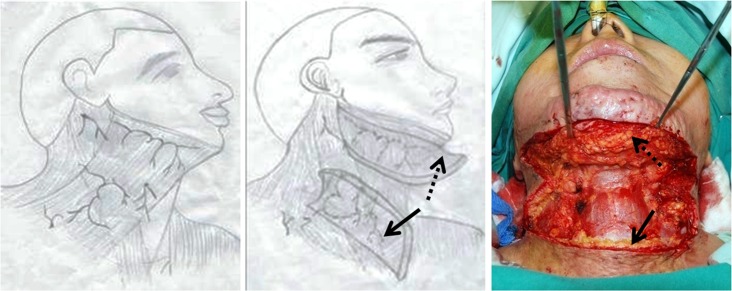
Platysma manifestation. *(Left)* blood supply of the platysma; *(Middle)* the two platysma flaps are elevated; *(Right)* The dotted arrow indicates the superiorly based muscular flap and the solid arrow indicates the inferiorly based flap.

Two platysma flaps formation (Fig 2). At the level of inferior border of thyroid cartilage, the platysma is divided into two muscle flaps—one is superiorly supplied by submental artery, another is inferiorly supplied by transverse cervical artery. The upper platysma flap was raised upward and fixed at the border of mandible to increase the fullness of the submental region. The lower platysma flap was raised downward and sutured to the scar section at the cervical-thoracic junction.

Skin grafting was performed to resurface the secondary defect, mainly with thick partial thickness skin graft. In case of limited skin donor site, acellular allo-dermal matrix (Beijing Jayya Life Biological Technology Co., Ltd.) combined with auto-epidermal graft were used. In the 2nd postoperative week, a tailored elastic bandage was used to compress the graft and the neck was splinted in an extended position for three to six months.

### Second stage treatment(3–6 months after first stage treatment)

Mental region scar resection. If there was lower lip eversion, the resection should extend to the border of vermilion for total reposition. Sometimes the eversion-derived lengthy lower lip deformity needed wedge excision. Commissurotomy is performed in case of microstomia.

Harvest of (para)scapular skin flap, mental region reconstruction. The size of 24 (para)scapular skin flaps ranged from 12 cm × 5.5cm to 18cm × 9.5cm., and the average width of the flap is 6.3±3.2 cm. Most donor sites (20/24) were closed directly. As recipient vessels the facial artery and vein were mainly used, although in some cases, they were narrowed and stiff due to posttraumatic inflammation. Size discrepancy existed between the branch of circumflex scapular vessels and recipient vessels may occur. If necessary, flaps with extensive subcutaneous tissue deposits underwent debulking after six months.

## Results

Except for two cases of partial skin graft necrosis that underwent secondary skin grafting and one case of flap congestion due to input-output imbalance (alleviated with bloodletting), all other patients completed the two stage treatment without difficulty. 24 patients were followed up from 4 to 60 months.

**Fig 3:**
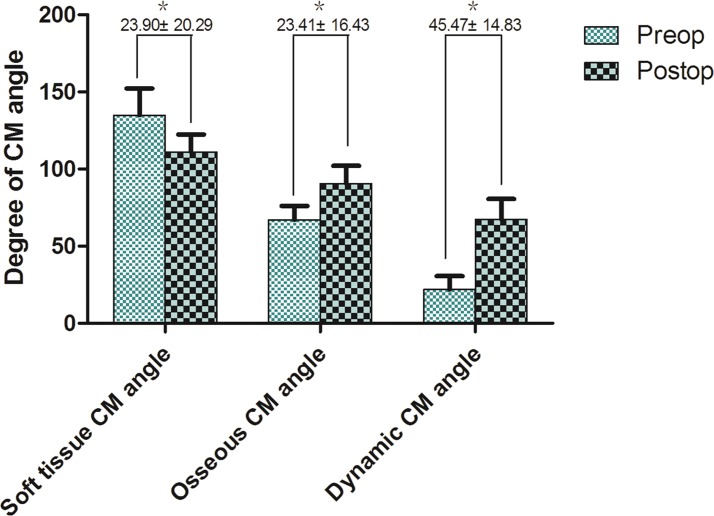
The significant change of three subtypes of CM angle after reconstruction (*, *p<0*.*05*). Statistical data shows soft tissue CM angle decreases after surgery that means the recovery of natural neck silhouette. Both osseous and dynamic CM angle increase after surgery that indicates patients achieve good functional results.

**Fig 4:**
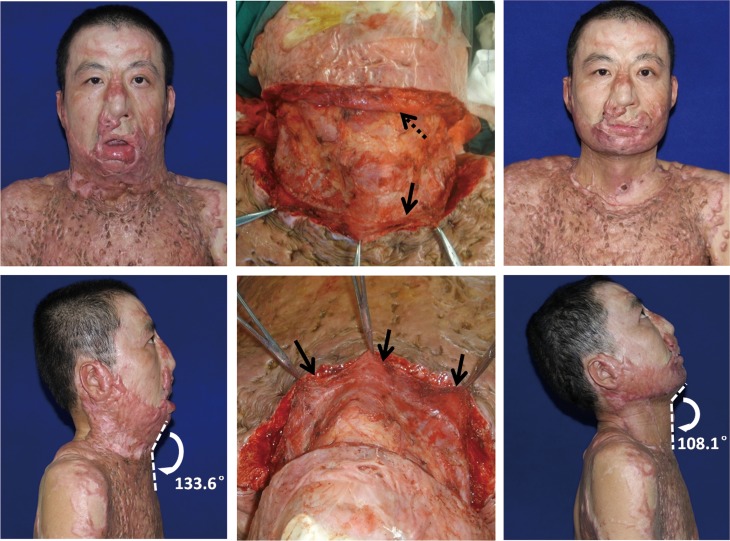
Male, 39 years old, one year after flame burn, underwent multiple skin grafting in other hospitals. *(Left column)* severe mentosternal contracture with soft tissue CM angle 133.6°. *(Middle column)* after release, platysma flaps were raised and turned *(arrows)*, partial-thickness skin graft sized 10 cm × 35 cm was harvested to resurface the secondary defect. *(Right column)* six months after surgery satisfactory reconstruction was achieved with soft tissue CM angle 108.1°. The patient refused the second-stage treatment due to financial concerns.

**Fig 5:**
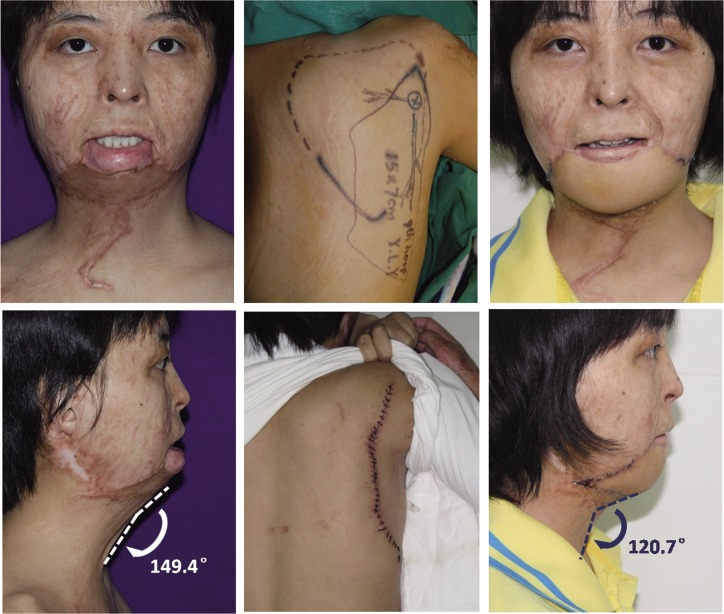
Female, 18 years old. 10 years after flame burn. *(Left column)* she underwent skin grafting two years ago, but the micrognathia remained with a wide soft tissue CM angle 149.4°. *(Middle column)* a parascapular skin flap sized 15cm × 7cm was designed to reconstruct her mental region and the donor site was closed directly. *(Right column)* satisfactory reconstruction results were achieved with soft tissue CM angle reduced to 120.7°.

**Fig 6:**
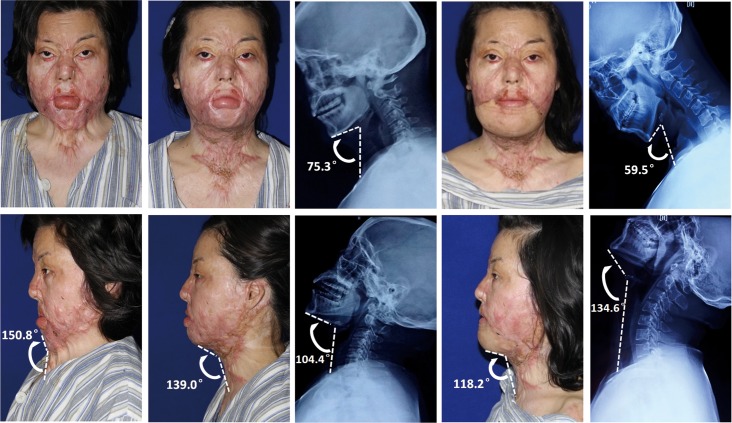
Female, 25 years old, 17 months after scalding during work, underwent skin grafting 6 times. *(Left column)* severe cervical contracture with soft tissue CM angle 150.8°. *(Second and middle column)* four months after the first stage treatment with partial thickness skin grafting (10cm×25cm), soft tissue CM angle was sharpened to 139.0°and functional CM angle reached to 29.1°. *(Fourth and right column)* six months after transplantation of a parascapular skin flap sized 7 cm × 15 cm to the mental region, a satisfactory result was achieved with further decrease of soft tissue CM angle to 118.2°and functional CM angle increased to 75.1°.

At the end of the third postoperative month after stage II treatment, soft tissue CM angle was significantly decreased from 135.0° ± 17.3° to 111.1° ± 11.3° (*p<0*.*05*), osseous CM angle significantly increased from 67.1° ± 9.0° to 90.5° ± 11.6° (*p<0*.*05*), and dynamic CM angle significantly increased from 21.9° ± 8.7° to 67.4° ± 13.1° (*p<0*.*05*) (Fig 3). Statistical analysis of three subtypes of CM angle’s difference before and after treatment shows this strategy achieved significant improvement. 22 (91.7%) patients got good functional result and natural neck silhouette by complete contracture removal (Figs 4, 5 and 6). They expressed satisfaction with the treatment. Recurrence of cervical contracture to different degree was observed in two patients who failed to wear elastic bandage and splint for three months.

## Discussion

### Cervicomental angle (CM angle)

Most of the existing evaluation systems for cervical contracture are descriptive [7, 8], while quantitative measurement and analysis is the prerequisite of objective comparison of different methods. A well-defined CM angle is a hallmark of normal cervical silhouette and can be measured quantitatively [10, 11]. The change of CM angles is an important manifestation of cervical contracture [7, 12]. The authors expand the traditional single CM angle to three subtypes:

1. Soft tissue CM angle, formed by surface marks of cervical soft tissue, is the static angle we usually refer to. It can also be regarded as aesthetic angle that will turn obtuse in contracture and even to nearly 180° in severe cases. Sometimes this index could be affected by multiple factors.
2. Osseous CM angle formed by bony marks reflects the long-term impact of cervical cicatrix on deep tissues. Its increase after treatment shows the effects of surgery on deep tissues.
3. Dynamic CM angle is the difference of osseous CM angles between in full cervical extension and in full cervical flexion, depicting the amplitude of cervical motion. We also call it the functional CM angle.

Quantitative measurement and comparison of these three subtypes of CM angles can provide a comprehensive and objective tool to help presurgical grading and postsurgical evaluation for severe cervical contracture.

### Platysma manipulation

In severe cervical contracture the platysma is also frequently involved. This muscle needs to be addressed if a full and lasting release is to be achieved. The upper part of platysma is supplied by submental artery and the lower part is supplied by transverse cervical artery [13, 14]. Based on the vasculature characteristics, the platysma can be horizontally divided into two muscle flaps. The upper muscle flap was reversed upward to be fixed to the margin of mandible. This procedure can increase the fullness of submental region, sharpen the soft tissue CM angle and avoid contracture recurrence simultaneously [11]. Furthermore, we turned the lower muscle flap downward to cover the scar section at the cervicothoracic junction to form smooth transition and facilitate the engraftment of skin graft. After the platysma manipulation, the neck was shaped slimmer with natural CM angle. This surgical technique provided not only functional reconstruction for cervical contracture, but also aesthetic improvement.

### Region-oriented treatment: skin flap vs skin graft

Kinds of skin flap were used for cervical contracture reconstruction. Axial flaps can be harvested from neighboring regions with a similar texture match, including transverse cervical artery-based flaps such as supraclavicular (island) skin flaps [3, 15], and internal mammary artery-based flaps such as deltopectoral flaps [4, 16]. In severe cases their sizes are often limited and can not be used for the extensive defects after release. Prior expansion could fabricate larger flaps and avoid skin grafting for the donor site [17]. However, for severe cervical contractures local procedures are often impracticable due to extensive adjacent scarring.

Free flaps such as groin flap [5], ALT [8], and scapular skin flaps [6, 18] have been reported for reconstruction of cervical contractures. A large free flap could resurface large defect, but at the cost of high donor site morbidity.

The neck region is a complex area with three-dimensional nature. It can be divided into three subunits: the anterior cervical region, submental region and mental region. In the strict sense the mental region belongs to the lower facial area, but for severe cervical contracture the mental region is always involved and needs to be addressed at the same time. Covering these subunits with one skin flap, especially thick flaps, often gives a less satisfactory neck contour with the loss of aesthetic CM angle to different extent.

Skin grafting still remains one of the most valuable tools in the burn reconstruction armamentarium. It possesses many advantages including easy manipulation, minor donor site injury and short treatment duration. Skin grafts are often the first choice to cover extensive defects created from scar excision and contracture release [1, 9, 19]. As for the flat anterior cervical region that is the biggest part of defect, and the submental region that consists of two layers of platysma after platysma flap turning, thick partial-thickness skin grafts can often achieve good functional and aesthetic results without contracture recurrence. In case of limited donor sites the acellular allo-dermis matrix combined with autologous epidermis grafting can be an alternative method [18].

The mental region possesses a protraction property and the importance of a strong chin is recognized as a well-defined CM angle [10, 12]. Because of the long-term contracture, especially incurred in childhood, the cervical scar will greatly retard the development of mandible. The results as micrognathia or retrognathia could be hardly altered by skin grafting in this zone. And even a minor contracture of the skin graft can lead to recurrence of deformity of the lower lip and the corner of the mouth. Similar to chin augmentation, a subtle yet dramatic improvement in the mental contour of patient can be made with en bloc skin flaps separately or in combination with sliding genioplasty.

(Para) scapular flaps are reliable with a consistent vascular pedicle of good length and large caliber. The color and structure of the back skin may provide a better match for mental region reconstruction than the flaps from extremities [6, 8, 18]. We have gained rich experience in (para)scapular flap harvest and application during past treatment. After the first stage of skin grafting in the neck area, the flap is used only for mental region reconstruction that can be less than 8 cm in width. The donor site can be closed directly with minimum morbidity.

### Staged reconstruction

In our opinion, different procedures for cervical subunits arranged in a staged sequence would be better. At first stage, treatment focuses on anterior cervical region and submental region. Full contracture release, platysma division then turnover and skin grafting will restore normal cervical motion and contour. Three to 6 months later, the second-stage treatment can be initiated. Without influence of anterior cervical and submental region contracture, the degree of micrognathia or retrognathia can be more precisely evaluated and reconstructed with free (para)scapular flaps.

Although staged strategy means longer treatment time and higher cost, a single-stage treatment including skin grafting and free flap transfer all at the same time will lead to additional risks. For example, skin grafting will need bolstering dressing and keep the neck in full extension, which will cause tension to the flap pedicle or site of blood vessels anastomosis. After free flap transplantation anticoagulant will need to be administered sometimes that can lead to a hematoma under the skin graft.

Based on statistical comparison of three subtypes of CM angle before and after treatment, the region-oriented and staged treatment strategy can achieve satisfactory functional and aesthetic results for severe cervical contracture, combining usage of both skin graft and skin flap while minimizing the donor site morbidity.

Similar to some publication describing techniques utilized to treat severe cervical contracture, our research employs retrospective design that has inherent defect when compared to prospective study. To evaluate and compare these techniques need more quantitative research. What's more, the rules of evidence based medicine should be followed [20]. High quality clinical trials, especially randomized controlled trials that currently is almost absent in this field will provide high quality evidence in forming treatment consensus and guidelines, which will help reconstructive surgeons make judicious decisions about the care of individual patients.
